# Targeting the COMMD4–H2B protein complex in lung cancer

**DOI:** 10.1038/s41416-023-02476-8

**Published:** 2023-11-01

**Authors:** Ming Tang, Joshua T. Burgess, Mark Fisher, Didier Boucher, Emma Bolderson, Neha S. Gandhi, Kenneth J. O’Byrne, Derek J. Richard, Amila Suraweera

**Affiliations:** 1 Queensland University of Technology (QUT), School of Biomedical Sciences, Centre for Genomics and Personalised Health at the Translational Research Institute, 37 Kent Street, Woolloongabba, QLD 4102 Australia; 2 Frazer Institute, Faculty of Medicine, The University of Queensland at the Translational Research Institute, 37 Kent Street, Woolloongabba, QLD 4102 Australia; 3 Princess Alexandra Hospital, 199 Ipswich Road, Woolloongabba, QLD 4102 Australia; 4 Department of Computer Science and Engineering, Manipal Institute of Technology, Manipal Academy of Higher Education, Manipal, Udupi, Karnataka 576104 India

**Keywords:** Non-small-cell lung cancer, Apoptosis

## Abstract

**Background:**

Lung cancer is the biggest cause of cancer-related deaths worldwide. Non-small cell lung cancer (NSCLC) accounts for 85–90% of all lung cancers. Identification of novel therapeutic targets are required as drug resistance impairs chemotherapy effectiveness. COMMD4 is a potential NSCLC therapeutic target. The aims of this study were to investigate the COMMD4-H2B binding pose and develop a short H2B peptide that disrupts the COMMD4-H2B interaction and mimics COMMD4 siRNA depletion.

**Methods:**

Molecular modelling, in vitro binding and site-directed mutagenesis were used to identify the COMMD4-H2B binding pose and develop a H2B peptide to inhibit the COMMD4-H2B interaction. Cell viability, DNA repair and mitotic catastrophe assays were performed to determine whether this peptide can specially kill NSCLC cells.

**Results:**

Based on the COMMD4-H2B binding pose, we have identified a H2B peptide that inhibits COMMD4-H2B by directly binding to COMMD4 on its H2B binding binding site, both in vitro and in vivo. Treatment of NSCLC cell lines with this peptide resulted in increased sensitivity to ionising radiation, increased DNA double-strand breaks and induction of mitotic catastrophe in NSCLC cell lines.

**Conclusions:**

Our data shows that COMMD4-H2B represents a novel potential NSCLC therapeutic target.

## Background

Lung cancer is the second most diagnosed cancer and the most common cause of death from cancer worldwide. In 2020, there was an estimated 2.2 million new lung cancers diagnosed and roughly 1.8 million deaths worldwide resulting from lung cancer, accounting for 11.4% of the total cancer burden [1]. Non-small cell lung cancers (NSCLC) are the most common type of lung cancer, comprising 85–90% of all lung cancers. NSCLC can be further classified into three types, adenocarcinoma (ADC), squamous cell carcinoma (SCC) and large cell carcinoma (LCC), contributing to roughly 40%, 30% and 10%, respectively of all lung cancers [2–4]. Radiotherapy and chemotherapy delivered sequentially or concurrently are important treatment strategies for treating NSCLC. However, these modalities are currently limited by primary or acquired resistance to cytotoxic drugs and radiation, leading to suboptimal outcomes [5]. While immunotherapy in the last few years has considerably changed the treatment landscape of NSCLC [6], nevertheless, dependent on stage and geographical differences, the 5-year survival rate for NSCLC is still 4–26% [7, 8]. Thus, there is still a need to identify novel anti-cancer therapeutic targets and therapies to improve the outcome, quality of life and symptom control of patients.

Every single day, cells are damaged by various exogenous and endogenous agents which can result in genomic instability or cancer. The DNA repair pathways of a cell function to maintain genomic stability and prevent the onset of cancer [9–11]. DNA double strand breaks (DSBs) are regarded as the among the most detrimental lesions to cells [10, 12]. Although DNA repair pathways function to prevent cancer, once a tumour has arisen, the same DNA repair pathways can be therapeutically exploited to kill cancer cells [13]. To this end, there are several inhibitors targeting DNA repair proteins and pathways under active development [14–17].

Copper metabolism gene MURR1 domain (COMMD) proteins are an emerging family of potential therapeutic targets in several cancers including lung, hepatocellular and renal cell carcinoma [18–24]. This family consists of 10 evolutionarily conserved members that have a characteristic C-terminal COMMD domain, and these proteins have been implicated in several biological functions including copper homoeostasis, NF-κB pathway regulation, protein trafficking and cell proliferation [25–27].

We have recently shown that COMMD4 is a promising NSCLC prognostic marker and anti-NSCLC therapeutic target [21]. Our study demonstrated that *COMMD4* transcript and protein expression was upregulated in NSCLC patients and this overexpression was prognostic for the ADC subtype. siRNA-mediated depletion of COMMD4 in NSCLC patient cell lines impaired proliferation and reduced the viability of these cells, with COMMD4 depletion resulting in mitotic catastrophe and NSCLC cell death [21]. We have further demonstrated that following the induction of DSBs, COMMD4 is critical in regulating the remodelling of chromatin around sites of DSBs. COMMD4 functions to sterically hinder the access of the E3 ligase complex, RNF20/40, to H2B dimerised to H2A, thus preventing ubiquitination of H2B and subsequent remodelling of the nucleosome. This suppression is lost when H2B is phosphorylated on S14, which causes COMMD4 to disassociate from H2B, giving access to RNF20/40, which is also in complex with COMMD4. Following displacement from H2B, COMMD4 binds to adjacent H2A, which orientates COMMD4 bound RNF20/40 towards H2B [28]. The initial recruitment of COMMD4 to the DSB site is human single stranded DNA binding protein 1 (hSSB1) dependent, specifically requiring hSSB1 to be phosphorylated either by the ATM (T117 [28, 29]) or DNA-PK kinases (S134 [28, 30]).

In this study we have determined the COMMD4-H2B binding pose and developed a specific 10-amino acid H2B peptide that binds to COMMD4, preventing H2B binding. Furthermore, we show that expression of this peptide in NSCLC cell lines mimics siRNA mediated COMMD4 depletion and induces mitotic catastrophe mediated cell death.

## Methods

### Molecular modelling

The structure of full-length H2B was adopted from the crystal complex structure of H2A-H2B (PDB ID: 2RVQ). The COMMD4 binding domain in H2B comprising residues 61–84 [28] was generated by deleting the rest of the amino acids in H2B. The N-terminal structure of COMMD4 was obtained by deleting the C-terminal domain of the full-length COMMD4 structure predicted by AlphaFold [31, 32]. Blind docking simulations of the COMMD4 binding domain in H2B with residues 61–84 to COMMD4 N-terminal domain were performed in ClusPro server [33–36] without applying any bias on the binding. Top 20 binding poses were obtained, and the corresponding complex structures were downloaded from the HADDOCK 2.4 server. By visualising the structure of the 20 downloaded complexes, a rough surface on COMMD4 that represents the highest chance to be the H2B binding region was determined. Then, HADDOCK 2.4 web server with HADDOCK default settings (https://wenmr.science.uu.nl/haddock2.4/) [37] was used to dock the full-length H2B to the N-terminal structure of COMMD4, where residues ranging from 61 to 84 were set for the COMMD4 binding sites in H2B. The H2B binding sites in COMMD4 were set as residues forming the potential H2B binding surface of COMMD4 predicted with ClusPro server. Top 20 binding poses predicted with HADDOCK 2.4 were downloaded.

The 20 obtained binding complexes of COMMD4-H2B from HADDOCK 2.4 server underwent three 100-ns-long independent equilibrium MD simulations starting from different atomic velocities. All MD simulations were carried out with the PMEMD module (CUDA version) of the AMBER 16 software suite with the ff14SB force field for proteins and TIP3P model for water molecules [38, 39]. Other conditions for the MD simulations were set similar to our previous published work [40–43]. Briefly, all solutes were solvated with an octahedral water box, making sure that the proteins were at least 12 Å away from edges of the water box. Periodic conditions were applied in all dimensions. Every system went through proper geometry optimisations before undergoing the equilibrium production runs. Subsequently, the optimised systems were heated properly for 1 ns in an NVT ensemble from 100 K to 310 K, followed by a 10-ns-long NPT annealing. Finally, MD simulations were carried out for each equilibrated system without any restraints, where the temperature was kept at 310 K by the Langevin thermostat with gamma_ln set to 2 [44] and the pressure was set at one atmosphere by the Berendsen barostat [45] and a pressure coupling constant of 1 ps. The Molecular Mechanics/Generalized Born Surface Area (MM/GBSA) binding free energies between H2B and COMMD4 were calculated by the MMPBSA.py script [46] in AmberTools 16 [47]. Trajectory visualisation and MM/GBSA binding free energies were used to determine the most possible binding pose of H2B to COMMD4. The binding pose with the lowest MM/GBSA binding free energy among the poses that were stable in the MD simulations was selected as predicted binding pose by molecular modelling. The schematic diagram of COMMD4-H2B was generated in PDBsum (https://www.ebi.ac.uk/thornton-srv/databases/pdbsum/).

### Circular dichroism

For Circular Dichroism (CD), H2BWT and H2BMUT1 peptides were resuspended at a concentration of 0.1 mg/ml in 50% 2,2,2-Trifluoroethanol (Sigma, T63002) with 10 mM Phosphate Buffer pH 7.2 and 50 mM sodium fluoride. The buffer solutions were filtered and degassed before diluting the peptides. The peptides were loaded into a 1 mm path-length quartz cuvette. CD spectra were measured at 20 °C in the far-UV from 250 to 190 nm with a Jasco J-1500 spectropolarimeter at a 0.1 nm interval with a 20 nm/min scan rate. All CD data represent an average of five scans, and the background was corrected with the buffer-matched diluent. The results were converted to the units of molar ellipticity vs. wavelength.

### Antibodies

The following primary antibodies were used in immunoblotting and immunofluorescence experiments; anti-COMMD4 (Abcam, ab115169), anti-β-actin (BD Biosciences, 612656), anti-FLAG (Sigma, F1804), anti-γH2AX (Abcam, ab26350), anti-H2B (Abcam, ab1790), anti-MDC1 (Abcam, ab11169) and anti-emerin (Cell Signaling 30853). For in vitro assays, we used the following IgG controls; normal mouse IgG (Sigma-Aldrich, N103), normal rabbit IgG (Cell Signaling, 2729) and normal sheep IgG (Sigma-Aldrich, 12–515). The following secondary antibodies were used for immunoblotting; IRDye^®^ 800CW Donkey anti-goat (LI-COR, 925–32214), IRDye^®^ 800CW Donkey anti-mouse (LI-COR, 926–32212) and IRDye^®^ 680CW Donkey anti-rabbit (LI-COR, 926–68073). We used the following secondary antibodies for immunofluorescence experiments; Alexa Fluor® 488 donkey anti-mouse (Life Technologies, A21202), Alexa Fluor® 594 donkey anti-rabbit (Life Technologies, A21207), Alexa Fluor® 488 donkey anti-rabbit (Life Technologies, A21206) and Alexa Fluor® 594 donkey anti-mouse (Life Technologies, A21203).

### Cell lines, cell culture, treatments and reagents

H1299, CRL5889 and H460 cells were cultured in Roswell Park Memorial Institute (RPMI) 1640 medium (Life Technologies) containing 10% foetal bovine serum (Thermo Fisher Scientific). Human bronchial epithelial cells (HBEC3-KT) were maintained in keratinocyte serum-free medium supplemented with epidermal growth factor and bovine pituitary extract (Life Technologies) [48]. The histology features and origin of all cell lines used have been previously described [21]. Cell lines were authenticated using STR profiling. Cells were grown in a humidified incubator at 37 °C/5% CO_2_ atmosphere. All cell lines were verified to be free of mycoplasma contamination.

Irradiations were done at room temperature using a ^137^Cs source (Gammacell 40 Exactor [MDS Nordion]; dose rate 1.1 Gy/min). Phalloidin-Atto 488 was purchased from Sigma-Aldrich and Hoechst 33342 was purchased from Thermo Fisher Scientific.

### Transfection of siRNA and expression constructs

For siRNA transfections, cells were transfected with negative control or COMMD4 siRNA #2 (CCAUGUCCCUCUCAGCAGA[dT][dT] MISSION^®^ siRNA, Sigma-Aldrich) using RNAiMax (Life Technologies) as previously described [21, 28] and samples were analysed 48 to 72 h post-transfection. The H2B wild-type (FLAG-H2BWT) and mutant (FLAG-H2BMUT1) expression constructs were generated with the 10-amino acid peptide and the corresponding mutant cloned into the expression vector pCDNA3.1/(+)-N-DYK containing an N-terminal FLAG tag and a C-terminal nuclear localisation signal (AAKRVKLDS) [49] (GenScript). A70K FLAG-COMMD4 expression construct was generated by mutating the corresponding COMMD4-FLAG construct [28] to harbour an A70K mutation (GenScript). Cells were mock-transfected, or transfected with FLAG-H2BWT/FLAG-H2BMUT1, or COMMD4-FLAG/A70K with FuGENE^®^ HD (Promega) using the manufacturer’s protocol and analysed 24 h post-transfection.

### Immunoblotting analyses

Immunoblotting was performed as previously described [50]. Briefly, cell pellets were lysed in ice-cold NP40 buffer (20 mM HEPES pH 8, 150 mM KCl, 10 mM MgCl_2_, 0.5 mM EDTA, 0.2 % NP40, 0.5, 5% glycerol, 1X protease and phosphatase inhibitor cocktail (ThermoFisher) and 1X Pierce Universal Nuclease for cell lysis (ThermoFisher)). 15–20 μg of cell lysates were separated on 4–12% Bis-Tris Plus Bolt precast gels (ThermoFisher) and immunoblotted with the antibodies indicated.

### Co-immunoprecipitations

Co-immunoprecipitations were performed as previously summarised, using ice-cold NP40 buffer [28]. Assays for co-immunoprecipitations were performed at 4 °C from HEK293T, transiently expressing the FLAG-H2B WT or MUT plasmid. Proteins were captured using anti-FLAG M2 Dynabeads (ThermoFisher) and washed three times in NP40 buffer prior to analysis.

### In vitro binding of COMMD4 and H2B

300 ng of recombinant GST-COMMD4 (Abnova, H00054939-P01) was incubated with 1 μg of recombinant H2B (NEB, M2505S) in NP40 buffer for 30 min at 4 °C. For competition experiments, 1 μg (or the amount specified) of the N-terminal biotin-tagged H2B peptide (GenScript) was pre-incubated with recombinant COMMD4 for 30 min at 4 °C prior to addition of recombinant H2B. Proteins were captured using Streptavidin Dynabeads (ThermoFisher) or Protein A Dynabeads (ThermoFisher), washed three times in NP40 buffer, resuspended in 4X Leammli sample buffer (250 mM Tris pH 6.8, 8% SDS, 4% glycerol, 0.02% bromophenol blue, 8% β-mercaptoethanol) and immunoblotted with the indicated antibodies.

### ELISA

ELISA was performed using NeutrAvidin High Binding Capacity Coated 96-Well Plates (Thermo Scientific, 15507) according to the manufacturer’s instructions. Briefly, to the appropriate well, added 100 μl of H2B peptide at 10 μg/ml for 2 h at room temperature. Washed each well three times with NP40 buffer, blocked the plate overnight with 4% BSA in NP40 buffer and the next day added different concentrations of recombinant GST-COMMD4 as shown, for 45 min at room temperature. Washed each well three times with NP40 buffer and added anti-COMMD4 antibody for 45 min at room temperature followed by the enzyme-linked secondary antibody for 30 min at room temperature followed by detection using SuperSignal ELISA Pico Chemiluminescent Substrate (Thermo Scientific, 37070).

### Cell viability assays

Clonogenic cell viability assays were performed as previous [28, 51]. In summary, post-transfection with control/COMMD4 siRNA or FLAG-H2BWT/FLAG-H2BMUT1 plasmid or mock-transfection, 400 cells were plated in each well of a six-well plate (Corning). The following day, the cells were irradiated and allowed to recover for 7–10 days. Every condition was plated in triplicate and performed three independent times. Dose–response curves were generated using Graphpad Prism 9 and data are represented as means ± SD.

A CellTiter-Glo 2.0 Luminescent assay (Promega Corporation) was carried out as previously described [52, 53]. Briefly, following transfection, 500 cells were plated into each well of a white-walled 384-well plate (Corning). The next day, cells were irradiated and 48 h post-irradiation, CellTiter-Glo 2.0 was added to each well according to the manufacturer’s recommendations. Luminescence was measured using a PHERAstar FSX detection system (BMG Labtech). Dose–response curves were generated with GraphPad Prism 9.

### Immunofluorescence microscopy and high-content microscopy

NSCLC and control cells transfected with siRNA or H2B plasmids were grown in optical glass bottom 96-well plates (Cellvis). Post-treatment, cells were pre-extracted with ice-cold extraction buffer (20 mM HEPES (pH 8), 20 mM NaCl, 5 mM MgCl_2_, 1 mM ATP, 0.5% NP40), to remove the soluble proteins and processed as previous [54]. Images were captured using a Delta Vision PDV microscope 100x/1.42 Oil objective (Applied Precision, Inc). All immunofluorescence figures were assembled using Adobe Photoshop. High-content microscopy was performed using the InCell Analyzer 6500 Imaging System (GE Healthcare Life Sciences). Images were analysed using the InCarta Image Analysis software (GE Healthcare Life Sciences) where a minimum of 50 nuclei were quantified per experiment.

### Immunofluorescence microscopy for evaluating mitotic catastrophe

NSCLC cells were grown in 96-well plates as above and stained with anti-emerin antibody and phalloidin. Mitotic catastrophe was scored as previous [21]. A minimum of 100 cells for each experimental condition were assessed to determine the percentage of aberrant nuclei.

### Apoptosis assays

The Annexin V-FITC apoptosis kit (United Bioresearch, ALX-850-020-K101, Enzo Life Sciences, Farmingdale, NY, USA) was used to measure viable and apoptotic cells, as previously described [21]. Briefly, transfected cells were resuspended in binding buffer containing annexin V-FITC. After incubation, cells were incubated with propidium iodide and flow cytometry was carried out using a cytoFLEX flow cytometer (Beckman Coulter, Brea, CA, USA). CytExpert 2.0 software was used for data acquisition and analysed using the FlowJo v10 software.

### Statistics and reproducibility

Data are presented as the mean ± SD from a minimum of three independent experiments. Statistical analyses were performed using a two-tailed non-paired Student’s *t*-test. The level of significance was set at *P ≤ 0.05 and **P ≤ 0.005. No statistical methods were used to predetermine sample size.

## Results

### Determining the COMMD4-H2B binding pose and designing and characterising the H2B peptides

**Fig. 1:**
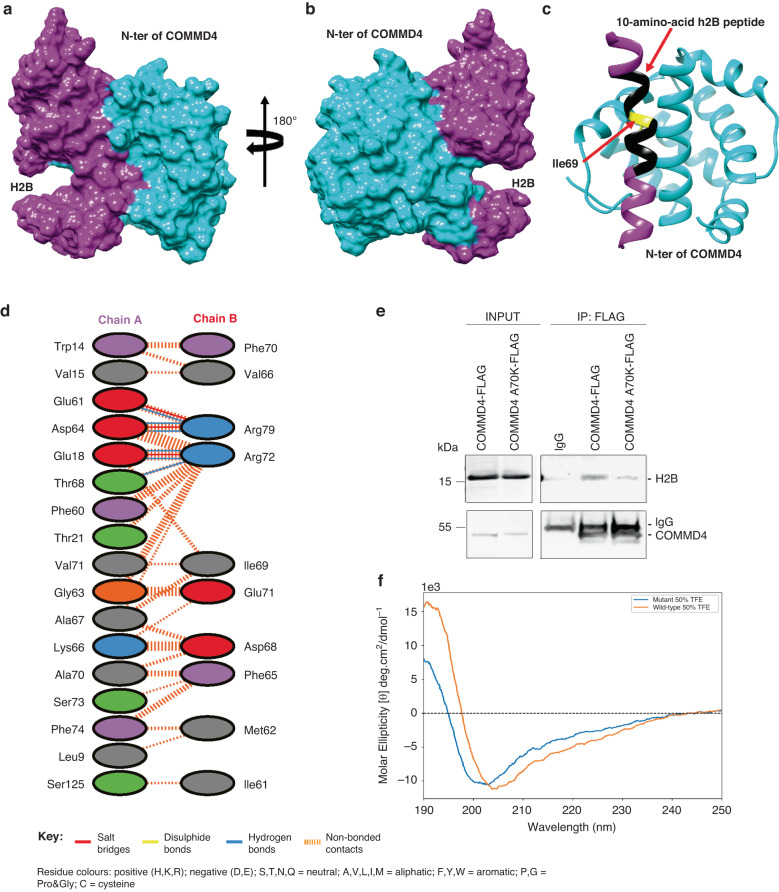
The binding pose of H2B and COMMD4. **a**, **b** Surface representations with different perspectives of the binding complex of COMMD4 N-terminal domain (cyan) and H2B (magenta) are shown. For the sake of clarity, surfaces of the N-terminal 35 residues in H2B that are not involved in binding to COMMD4 are not displayed in (**a**) and (**b**). **c** Shown is the ribbon representation of COMMD4 N-terminal domain (cyan) in complex with a H2B region (magenta) that contributes to the major binding of H2B to COMMD4. The identified 10-amino acid H2B peptide incorporating the key amino acids dominating the binding of H2B to COMMD4 is shown in black. The residue Ile69 mutated in H2BMUT is highlighted in yellow (**d**). Shown is the schematic diagram of the interactions between the two proteins displayed in (**c**). The number of H-bond lines between any two residues indicates the number of potential hydrogen bonds between them. For non-bonded contacts, which can be plentiful, the width of the striped line is proportional to the number of atomic contacts. **e** A FLAG pull-down experiment demonstrating that compared to COMMD4-FLAG expressing cells, cells expressing A70K-FLAG mutant showed reduced binding with H2B. IgG shows the loading. **f** CD spectra of the H2BWTpep (wild-type) and H2BMUT1pep (mutant) peptides in 50% TFE. Both peptides present peak ellipticity maxima at 190 nm, with H2BWTpep being more pronounced. The H2BWTpep presents a peak minimum at 204 nm, while the H2BMUT1pep minimum shifts towards 202 nm. These spectra indicate mixed helical and denatured/extended conformations, with H2BWTpep possessing a more helical secondary structure than H2BMUT1pep, which possesses little helical structure even in 50% TFE.

Our published data showed that COMMD4 functions in an intricate manner with other proteins to control chromatin remodelling at sites of DSBs [28]. Our previous peptide mapping experiments showed that H2B interacts with the N-terminal domain of COMMD4 [28], via a helical region comprising amino acids ranging from 61 to 84 [28]. To refine this region further, we sought to further predict the COMMD4-H2B binding pose (Fig. 1a–c) by performing comprehensive molecular docking and molecular dynamics (MD) simulations. This revealed that H2B binds tightly to the N-terminal domain of COMMD4 with a large and contentious interface (Fig. 1a, b). Figure 1d demonstrates a schematic diagram of the N-terminal domain of COMMD4 (Chain A) and a helical region of H2B (Chain B) that is the major COMMD4 binding region. This schematic indicates three salt bridges, six hydrogen bonds and 96 non-bonded interactions potentially between the two protein chains. By closely examining the schematic diagram and the 3D binding pose of COMMD4-H2B complex, we found that our modelled binding pose agrees with our previously published mutations in COMMD4 that contributed to its binding to H2B (Fig. 1d) and reduced their binding capability to H2B protein (Supplementary Table 2 in ref. [28]). This suggests the reliability of our modelling binding pose. To further validate our modelling binding pose, we explored the effect of A70K mutation in COMMD4 on its binding strength to H2B. A FLAG pull-down showed that compared to COMMD4-FLAG, A70K COMMD4-FLAG mutant showed reduced binding with H2B, demonstrating that residue Ala70 in COMMD4 is a potential binding site for H2B (Fig. 1e). This in turn justifies the reliability of the binding pose obtained from our modelling (Fig. 1c, d).

Based on the experimentally validated binding pose and the schematic diagram of the COMMD4-H2B complex, we identified a 10-amino acid wild-type H2B peptide, H2BWTpep shown in black in Fig. 1c (SFVNDIFERI), incorporating the key amino acids in H2B that are responsible for its interaction with COMMD4. We further designed a corresponding mutant peptide, H2BMUT1pep (SFVNDKFERI), which eliminated its COMMD4-binding capability.

Since peptides containing ten amino acids are relatively short, there is a possibility that the two designed H2B peptides may not form a helical structure when truncated from the full-length H2B protein. Therefore, we used CD spectrum to characterise the secondary structure of the two designed H2B peptides. Our CD spectra detected helicity for both H2B peptides, with the H2BWTpep showing more helicity than H2BMUT1pep (Fig. 1f).

### Validating the binding of H2B peptides to COMMD4 in vitro

**Fig. 2:**
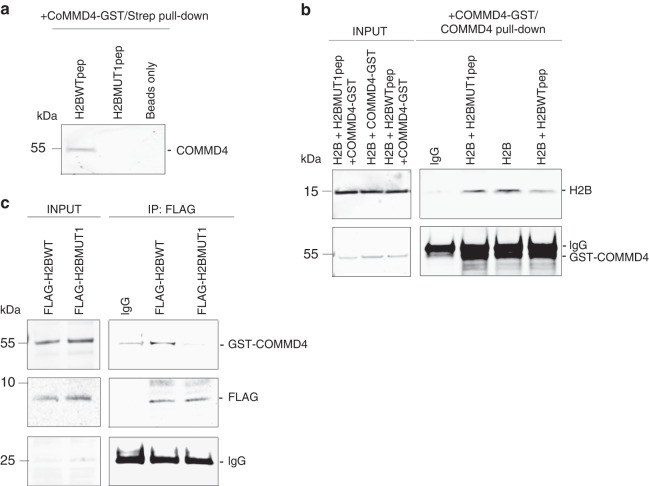
In vitro binding of the H2B peptide. **a** In vitro binding of biotin-tagged H2B 10-amino acid peptide (H2BWTpep) and H2BMUT1pep (mutation of key amino acid) with COMMD4. Beads only lane is the negative control. **b** A COMMD4 pull-down demonstrating that pre-incubation with the 10-amino acid H2B peptide (H2BWTpep) inhibits the COMMD4-H2B interaction, while pre-incubation with the mutant peptide (H2BMUT1pep) which does not bind to COMMD4, maintains the COMMD4-H2B interaction. INPUT shows the loading. **c** Binding of overexpressed H2BWTpep (FLAG-H2BWT) and H2BMUT1pep (FLAG-H2BMUT1) with GST-COMMD4. FLAG-H2BWT and FLAG-H2BMUT1 were immunoprecipitated from HEK293T cells and the co-eluting proteins were immunoblotted with antibodies against GST-COMMD4 and FLAG. IgG heavy chain shows the loading.

To support predicted models, we next designed peptides to perform mapping. We utilised the H2BWTpep, along with the corresponding mutant peptide, H2BMUT1pep, with a critical amino acid, as predicted by molecular dynamics mutated to eliminate the COMMD4-binding capability, in pull-down assays. Biotin-tagged H2B peptides designed as above were used in a purified system to pull-down recombinant COMMD4. Further to our previous data [28] and Fig. 1, in vitro streptavidin pull-down experiments confirmed that H2BWTpep is sufficient to bind to COMMD4 (Fig. 2a), while its corresponding mutant, H2BMUT1pep, is not capable of binding to COMMD4 (Fig. 2a). Next, COMMD4 pull-down experiments showed that pre-incubation of H2BWTpep with recombinant COMMD4 diminished the COMMD4-H2B interaction (Fig. 2b), while pre-incubation with H2BMUT1pep did not affect the COMMD4-H2B interaction. To confirm the specific binding between H2BWTpep and COMMD4, we performed an ELISA (Fig. S1a) and obtained an EC50 of 70 nM. Expression of H2BWTpep and its corresponding mutant, H2BMUT1pep, from an N-terminal FLAG expression vector recapitulated the result of Fig. 2a, showing that the vector expressing the 10-amino acid wild-type H2B peptide (FLAG-H2BWT) interacted with COMMD4, while its corresponding mutant (FLAG-H2BMUT1) was not capable of binding COMMD4 (Fig. 2c). Furthermore, the specificity of FLAG-H2BWT and FLAG-H2BMUT1 for COMMD4 was additionally demonstrated by initially depleting COMMD4 from cells using control and COMMD4 siRNA and subsequently overexpressing FLAG-H2BWT and FLAG-H2BMUT1 from these cells. A FLAG pull-down demonstrated that FLAG-H2BWT binding to COMMD4 was only possible in control siRNA-depleted cells (Fig. S1b).

### COMMD4 function is critical for survival of NSCLC cells following exposure to ionising radiation (IR)

**Fig. 3:**
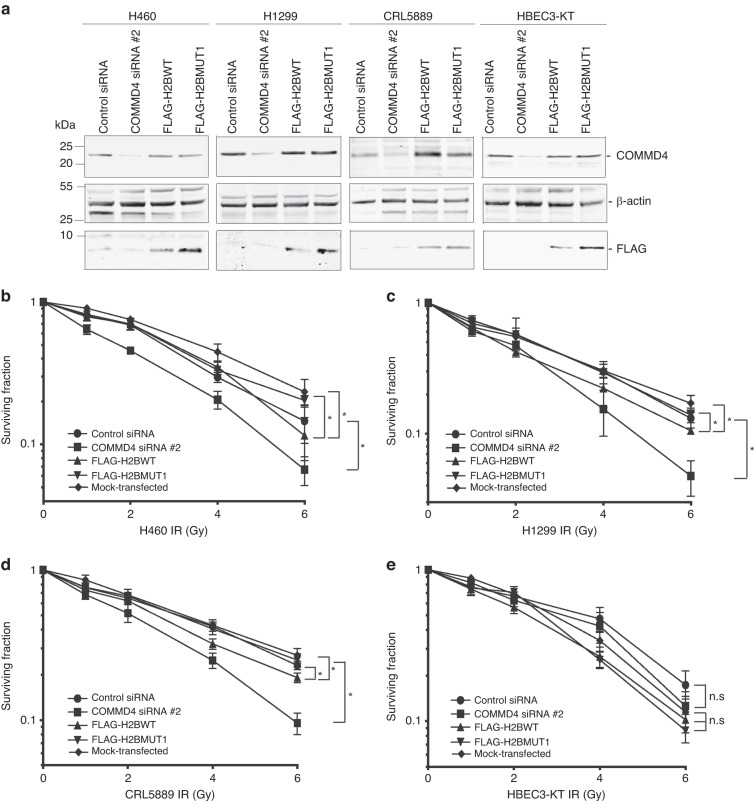
Viability of NSCLC cells post-transfection and IR exposure. **a** Immunoblot showing COMMD4 levels in NSCLC and control HBEC3-KT cells transfected with control siRNA or COMMD4 siRNA #2 and FLAG-H2BWT or FLAG-H2BMUT1. β-actin shows the loading and FLAG shows expression of the H2B constructs. **b**–**e** Clonogenic cell viability assays in H460, H1299 and CRL5889 NSCLC cells and HBEC3-KT cells transfected with control or COMMD4 siRNA #2 and FLAG-H2BWT or FLAG-H2BMUT1 or mock-transfected and treated with varying doses of irradiation (IR). Asterix (*) denotes *P* < 0.05, n.s; not significant. Error bars represent mean ± SD from three independent experiments.

COMMD4 depleted cells are hypersensitive to DNA damaging agents that cause DSBs [21, 28]. Since H2BWT bound to COMMD4 and inhibited the COMMD4-H2B interaction (Fig. 2), we next determined whether overexpression of this peptide in NSCLC and control human bronchial epithelial cells, HBEC3-KT, can mimic COMMD4 siRNA depletion. The FLAG-tagged H2BWT and H2BMUT1 overexpressed proteins (Fig. 2c) overexpressed from NSCLC and HBEC3-KT cells were ~7 kDa in size (Fig. 3a). We did not observe any effect of our peptide on COMMD4 expression. Clonogenic cell viability assays were next performed to determine the viability of HBEC3-KT and NSCLC cells with and without expression of our peptide. As we have previously shown [21], we observed that COMMD4 siRNA treated NSCLC cells were hypersensitive to IR compared to control siRNA treated cells (Fig. 3b–d). Interestingly, inhibiting the COMMD4-H2B interaction with the H2B WT peptide (FLAG-H2BWT) resulted in a similar phenotype to COMMD4 siRNA depletion, albeit less pronounced, where hypersensitivity to IR was observed relative to cells expressing the mutant peptide (FLAG-H2BMUT1) (Fig. 3b–d) or mock-transfected cells. As previously observed [21], HBEC3-KT cells were neither hypersensitive to IR after COMMD4 siRNA depletion, or after FLAG-H2BWT transfection (Fig. 3e). We additionally performed a luminescent cell viability assay in NSCLC and control cells to determine the hypersensitivity of FLAG-H2BWT, FLAG-H2BMUT1 and mock-transfected cells to IR. As observed with the clonogenic viability assays (Fig. 3), FLAG-H2BWT transfected NSCLC cells were hypersensitive to IR compared to FLAG-H2BMUT1 and mock-transfected cells. HBEC3-KT cells were not hypersensitive as expected (Fig. S2).

### Inhibition of the COMMD4-H2B interaction leads to the increased induction of DSBs

**Fig. 4:**
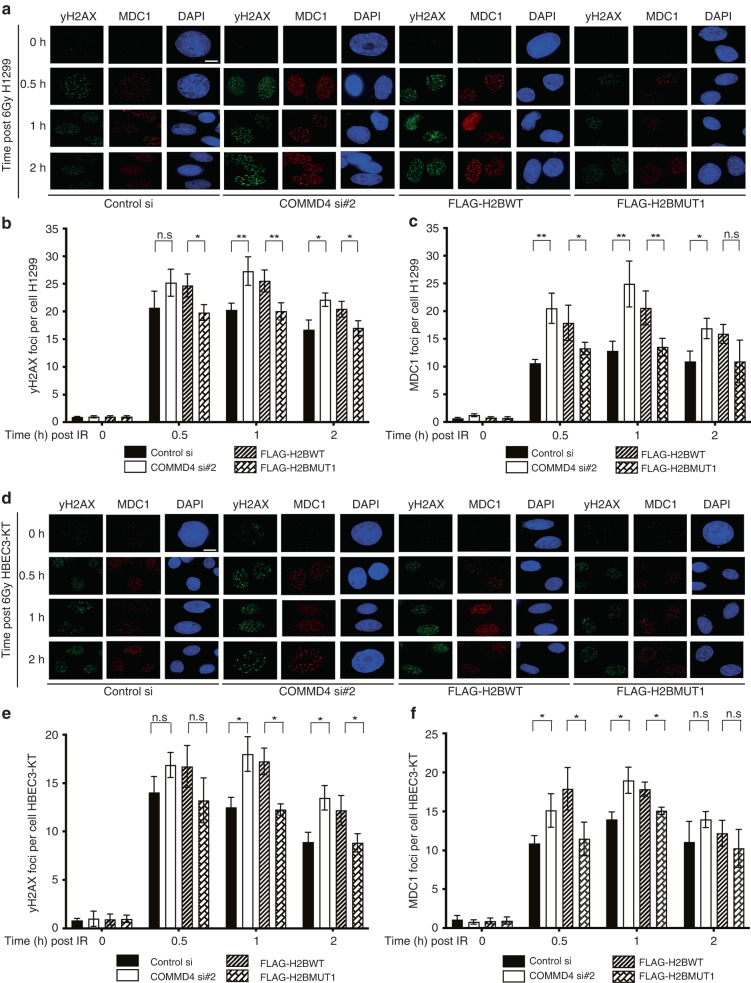
Induction of DSBs in NSCLC and control cells. **a** Immunofluorescence staining of H1299 NSCLC cells transfected with control or COMMD4 siRNA #2 and FLAG-H2BWT or FLAG-H2BMUT1 is shown. 48–72 h post-siRNA transfection and 24 h post-FLAG H2B transfection, cells were treated with 6 Gy IR and the recovery time points (h) post-IR treatment is shown. **b**, **c** Plot of γH2AX and MDC1 foci numbers for (**a**). **d** Immunofluorescence staining of HBEC3-KT control cells as in (**a**). **e**, **f** Plot of γH2AX and MDC1 foci numbers for (**d**). n.s; not significant, *; *P* < 0.05 and **; *P* < 0.005. Error bars represent mean ± SD from three independent experiments where 50 cells were quantified per condition. γH2AX (green), MDC1 (red) and DAPI shows the nucleus. Scale bar denotes 5 μm.

We have previously demonstrated that COMMD4 is required for the repair of DSBs [28] and COMMD4 depleted cells have increased γH2AX (an indirect marker of DSBs [55]) and MDC1 [56] foci, indicative of more DSBs and larger repair foci [28]. Here we examined whether cells expressing the H2B WT peptide had increased foci after inhibition of the COMMD4-H2B interaction. Immunofluorescence was used to visualise γH2AX and MDC1 foci formation in NSCLC and control HBEC3-KT cells transfected with control/COMMD4 siRNA or FLAG-H2BWT/FLAG-H2BMUT1 plasmids. Here we observed that cells depleted of COMMD4 had significantly more γH2AX and MDC1 foci (Fig. 4 and Fig. S3 and S4) from 0.5 to 2 h post-IR. HBEC3-KT cells also exhibited increased DNA repair foci in COMMD4 depleted and FLAG-H2BWT transfected cells, however, these foci numbers were less than in NSCLC cells (Fig. 4). Interestingly, cells transfected with FLAG-H2BWT and not FLAG-H2BMUT1, displayed a similar phenotype to COMMD4 siRNA depleted cells and had significantly more DNA repair foci, indicative of unrepaired DSBs. These data highlight that the disruption of the COMMD4-H2B interaction in cells results in a similar phenotype as COMMD4 siRNA depletion.

### Expression of the H2B peptide leads to the induction of mitotic catastrophe and apoptosis in NSCLC cells

**Fig. 5:**
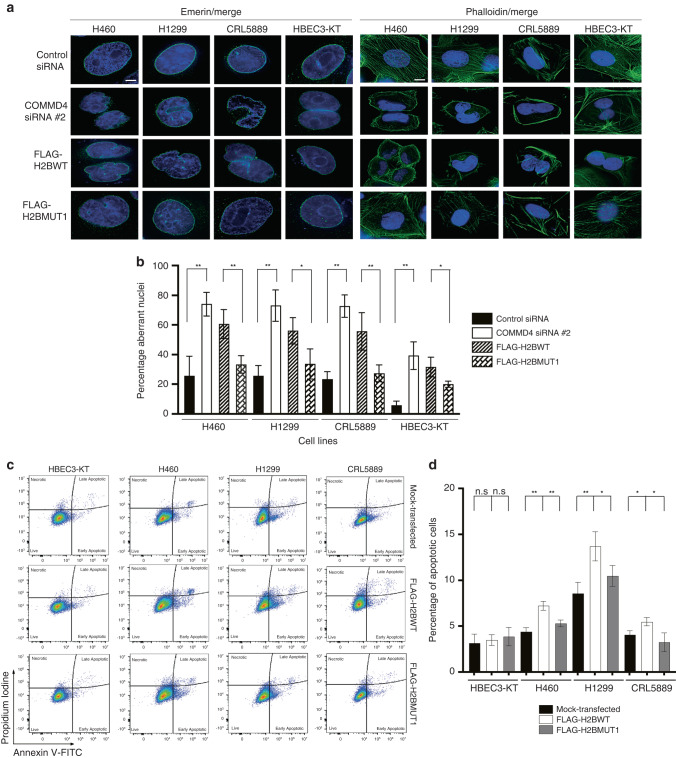
Induction of mitotic catastrophe and apoptosis in NSCLC cells. **a** Emerin and phalloidin staining respectively in three NSCLC and control HBEC3-KT cells. DAPI shows the nucleus and emerin and phalloidin staining are shown in green. **b** Quantification of the percentage aberrant nuclei for (**a**). Asterix (*) denotes *P < 0.05 and ** *P* < 0.005 from 100 cells counted. Error bars represent mean ± SD. Scale bar denotes 5 μm for Emerin and 15 μm for Phalloidin. (**c**). Flow cytometry analyses of apoptotic cells transfected with FLAG-H2BWT, FLAG-H2BMUT1 or mock-transfected. The live cells were stained with Annexin V-FITC and propidium iodide and analysed by flow cytometer. Live, necrotic and apoptotic cells are shown. **d** The percentage apoptotic cells are shown, which was calculated from by adding the early and late apoptotic cells. *P < 0.05, **P < 0.005, n.s; not significant. Error bars represent mean ± S.D from three independent experiments.

We previously demonstrated that NSCLC cells depleted of COMMD4 undergo mitotic catastrophe [21]. As we observed that NSCLC cells expressing FLAG-H2BWT displayed hypersensitivity to IR and increased γH2AX and MDC1 foci after IR exposure (Figs. 3 and 4), we sought to determine whether expression of this peptide could also increase mitotic failure. As previously determined, in this study, we used two markers to determine mitotic catastrophe: emerin staining of the nuclear envelope and phalloidin staining of the F-actin cytoskeleton [57–59]. Similar to COMMD4 depleted NSCLC cells, transfection of NSCLC cells with FLAG-H2BWT resulted in significantly more aberrant nuclei, indicative of mitotic catastrophe induction, relative to FLAG-H2BMUT1, mimicking COMMD4 siRNA depletion in these cells (Fig. 5a, b). As previous [21], HBEC3-KT cells also underwent mitotic catastrophe, but to a lesser extent than the NSCLC cell lines (Fig. 5a, b). We subsequently measured apoptosis by staining for Annexin V and propidium iodide via flow cytometry in FLAG-H2B WT, FLAG-H2BMUT1 and mock-transfected control and NSCLC cells (Fig. 5c, d). As expected, HBEC3-KT transfected cells showed no significant induction of apoptosis. H460, H1299 and CRL5889 NSCLC cells transfected with FLAG-H2BWT, showed significantly increased early and late apoptosis compared to FLAG-H2BMUT1 and mock-transfected cells (Fig. 5c, d), with FLAG-H2BWT transfection mimicking COMMD4 siRNA depletion.

## Discussion

COMMD4 functions to maintain genomic stability by regulating chromatin remodelling at DSBs. At sites of DSBs, COMMD4 binds to and protects H2B from monoubiquitination by the E3 ligase complex, RNF20/40 [28]. The monoubiquitination of H2B is required for the efficient repair of DSBs [60–63]. H2B monoubiquitination has been shown to disrupt chromatin compaction and inter-fibre interactions, leading to an open and accessible chromatin fibre [64]. In line with this, cells depleted of COMMD4 demonstrate excessive elongation of remodelled chromatin and defective repair of DSBs [28]. Here, we report the COMMD4-H2B binding pose predicted from comprehensive molecular docking and MD simulations, followed by validation with site-directed mutagenesis experiments. Based on the predicted and validated binding information, we identified a 10-amino acid helical region of H2B that incorporates the key amino acids responsible for its binding to COMMD4. The specific direct binding of the designed H2B peptide to COMMD4 was validated by in vitro direct binding experiments, ELISA experiments with an EC50, and reduced COMMD4 binding with a H2B peptide mutant, as a negative control. We subsequently characterised the secondary structure (helicity) of the two designed H2B peptides, with the peptide mutant showing less helicity. The severe helicity observed for H2BWTpep indirectly suggests that it mimics the full-length H2B protein in binding to COMMD4, thus inhibiting the binding of H2B protein to COMMD4. Notably, the less helicity of H2BMUT1pep than H2BWTpep might play a role in its unbinding activity to COMMD4. However, we believe that this unbinding can be purely induced by the unfavourable interactions and steric clashes between the mutant peptide and COMMD4, as the mutation site (Ile69) on H2B is fully buried in the COMMD4-H2B complex forming strong hydrophobic interactions with residues in COMMD4 and the sidechain of Lys, which is much longer than that of Ile and is very hydrophilic. We further investigated the inhibitory activity of the peptide by examining whether it can mimic COMMD4 siRNA depletion in cells. COMMD4 is overexpressed in NSCLC and this overexpression is associated with poor prognosis for ADC patients. Depletion of COMMD4 in NSCLC patient cell lines using two independent siRNA sequences has previously been shown to significantly impair proliferation and viability of these cells and lead to the induction of mitotic catastrophe [21]. We suggest that loss of functional COMMD4 in NSCLC cell lines results in uncontrolled chromatin remodelling due to their inherent genetic instability, a universal hallmark of cancer [21, 28].

Here, we cloned the H2B peptide (FLAG-H2BWT) and a corresponding mutant (FLAG-H2BMUT1), unable to bind COMMD4, into a FLAG expression construct. We next established whether NSCLC cells transfected with FLAG-H2BWT recapitulated COMMD4 depletion due to inhibition of the COMMD4-H2B interaction and inability to function after the induction of DNA double strand breaks. H460 (large cell carcinoma), H1299 (adenocarcinoma), CRL5889 (squamous cell carcinoma) NSCLC representing each subtype and HBEC3-KT (human bronchial epithelial cells) were initially transfected with siRNA or the H2B peptide expression construct and subjected to clonogenic cell viability assays post exposure to IR. We observed that similar to cells depleted of COMMD4, cells overexpressing FLAG-H2BWT were hypersensitive to IR, highlighting that FLAG-H2BWT overexpression mimics COMMD4 depletion in NSCLC cells. This suggests that functional COMMD4 is required for the survival of NSCLC cells after irradiation. We next examined whether we observed increased induction of DNA repair foci post-transfection of NSCLC cells with siRNA or FLAG-H2BWT. Significantly more γH2AX and MDC1 foci persisted in cells transfected with COMMD4 siRNA or the FLAG-H2BWT plasmid, indicating the presence of more DSBs upon disruption of the COMMD4-H2B interaction.

Since NSCLC cells depleted of COMMD4 undergo mitotic catastrophe and COMMD4 depletion may lead to chromothripsis [21], we investigated whether NSCLC and control cells transfected with COMMD4 siRNA or the FLAG-H2BWT plasmid have a similar phenotype. Emerin staining of the nuclear envelope and phalloidin staining of the F-actin cytoskeleton showed that similar to COMMD4 depleted cells, NSCLC cells transfected with FLAG-H2BWT experienced significantly more mitotic catastrophe than control cells. Transfection with FLAG-H2BWT specifically resulted in significantly more apoptosis of NSCLC cells, further confirming the potential of targeting the COMMD4-H2B complex in NSCLC.

Taken together, we have characterised the binding pose of the COMMD4-H2B protein complex and have identified a 10-amino acid peptide that directly binds to COMMD4, preventing COMMD4-H2B binding. By inhibiting the COMMD4-H2B interaction with this peptide, we can mimic COMMD4 siRNA depletion in NSCLC cells. NSCLC cells transfected with the FLAG-H2BWT plasmid are sensitive to irradiation, have increased DSBs and undergo mitotic catastrophe induced apoptosis. Our data demonstrate that the COMMD4-H2B protein complex is critical for maintaining genomic stability. COMMD4 plays a critical role in cancers, functioning to allow genetically unstable cancer cells to survive by limiting excessive remodelling of chromatin and dampening DNA damage-induced cell signalling and apoptosis [21, 28]. We suggest that inhibiting the COMMD4-H2B interaction, with a drug, could represent a therapeutic option for genetically unstable NSCLC. Protein-protein interactions (PPI) are vital in regulating signalling pathways in many biological processes. PPI-focused drugs are becoming an emerging field in drug discovery and peptidomimetics have been applied as a strong tool to inhibit PPI. Peptide drugs account for 5% of the global pharmaceutical market, with global sales exceeding US$50 billion in 2019 [65]. Furthermore, we suggest that targeting COMMD4-H2B with a drug may potenitally act as a radiosensitiser, enhancing the efficacy of radiotherapy for NSCLC patients.

### Supplementary information

Supplementary figures and legends

Original uncropped western blots

## Data Availability

All data presented in this study are included within the paper and its Supplementary files. The data that support the findings of this study are available upon request through the corresponding authors.
