# Supplementary material for: Targeting the COMMD4–H2B protein complex in lung cancer
Source: Br J Cancer. 2023 Nov 1;129(12):2014–24. doi: 10.1038/s41416-023-02476-8 (PMC10703884; doi:10.1038/s41416-023-02476-8)
Supplement: Supplementary file 1 — Supplementary figures and legends [file 41416_2023_2476_MOESM1_ESM.pdf]

Supplementary Figures and Legends

a

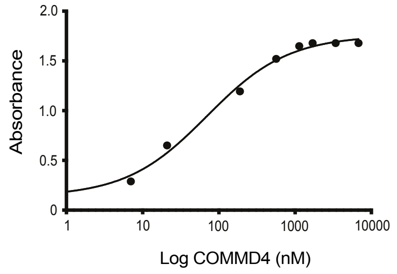

b

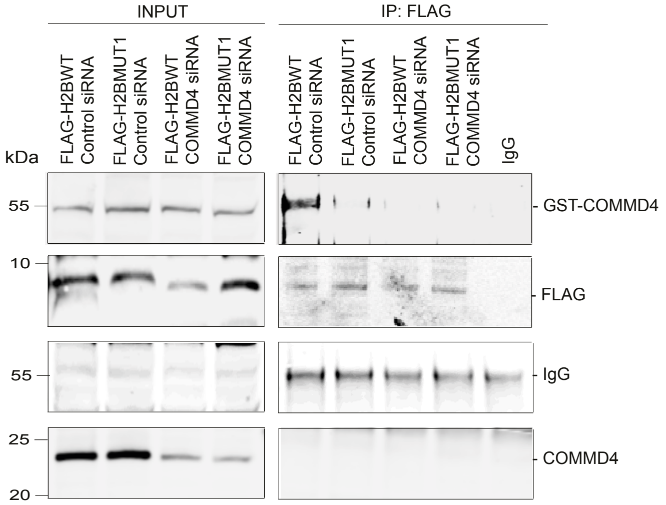

Fig. S1. Confirming the COMMD4 and H2B binding pose. (a). An ELISA showing the binding of H2BWTpep and recombinant COMMD4. An EC50 of 70 nM for COMMD4 is shown. **(b)**. The binding of overexpressed FLAG-H2BWT and FLAG-H2BMUT1 with GST-COMMD4 in control and COMMD4 siRNA depleted cells. COMMD4 levels are shown, and IgG heavy chain shows the loading.

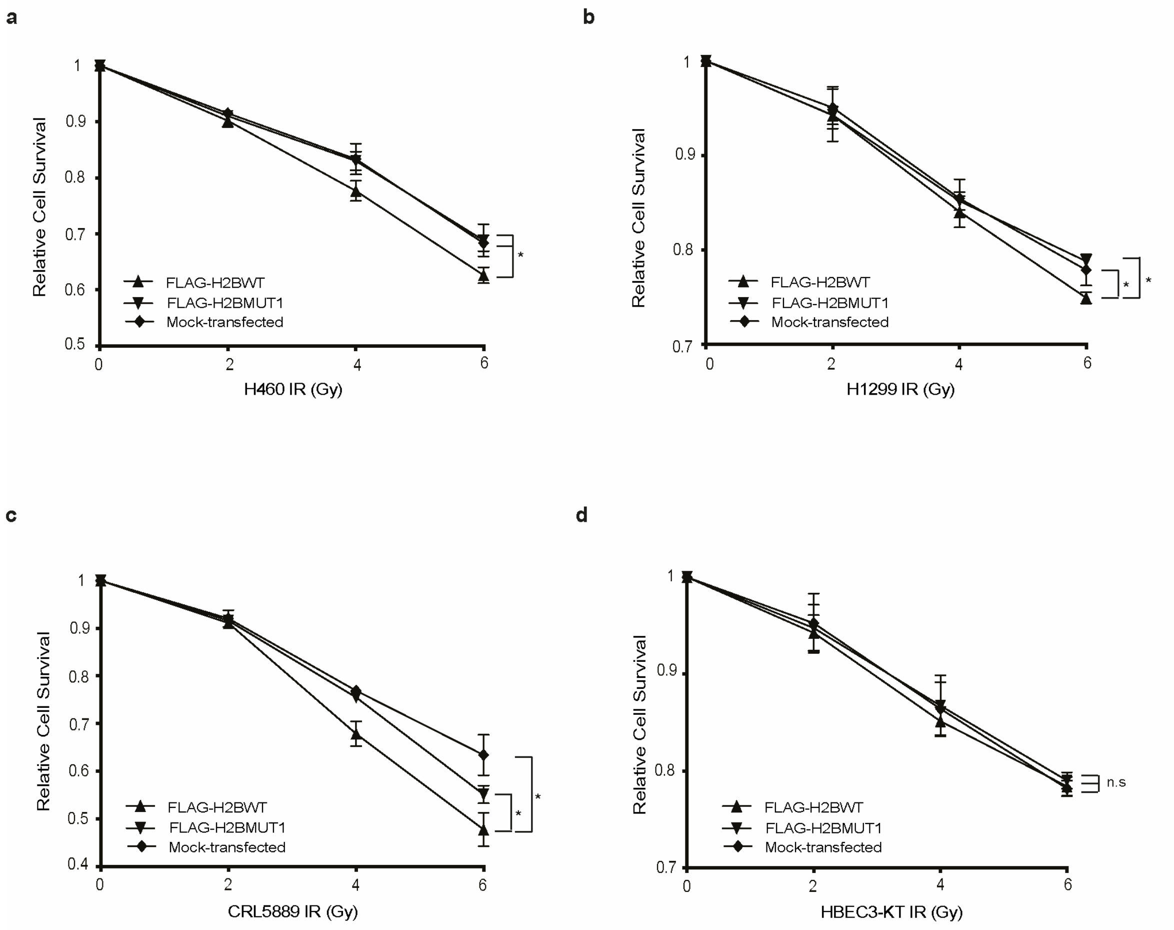

Fig. S2. Viability of NSCLC cells. (a-d). Luminescent cell viability assay of H460, H1299 and CRL5889 NSCLC cells and HBEC3-KT cells, transfected with FLAG-H2BWT, FLAG-H2BMUT1 or mock-transfected and treated with varying doses of IR is shown. Asterix (*) denotes $P < 0.05$, n.s; not significant. Error bars represent mean ± SD from three independent experiments.

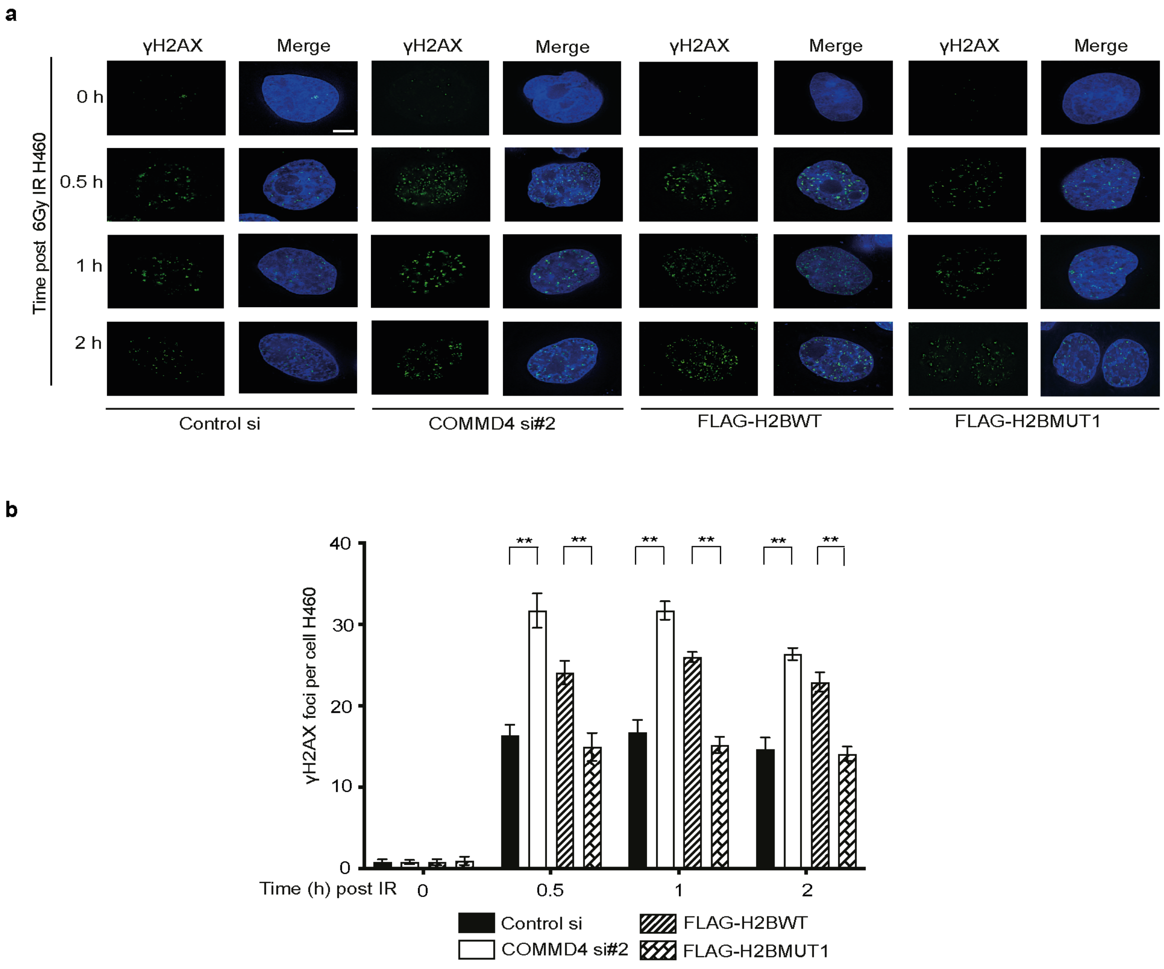

Fig. S3. Induction of γH2AX foci in H460 cells. (a). Immunofluorescence staining of H460 NSCLC cells transfected with control or COMMD4 siRNA #2 and FLAG-H2BWT or FLAG-H2BMUT1, treated with 6 Gy IR followed by recovery at time points shown. **(b)**. Plot of γH2AX foci numbers for **(a)**. **; $P < 0.005$. Error bars represent mean ± SD from three independent experiments where 50 cells were quantified per condition. γH2AX is shown in green and DAPI shows the nucleus. Scale bar denotes 5 μm.

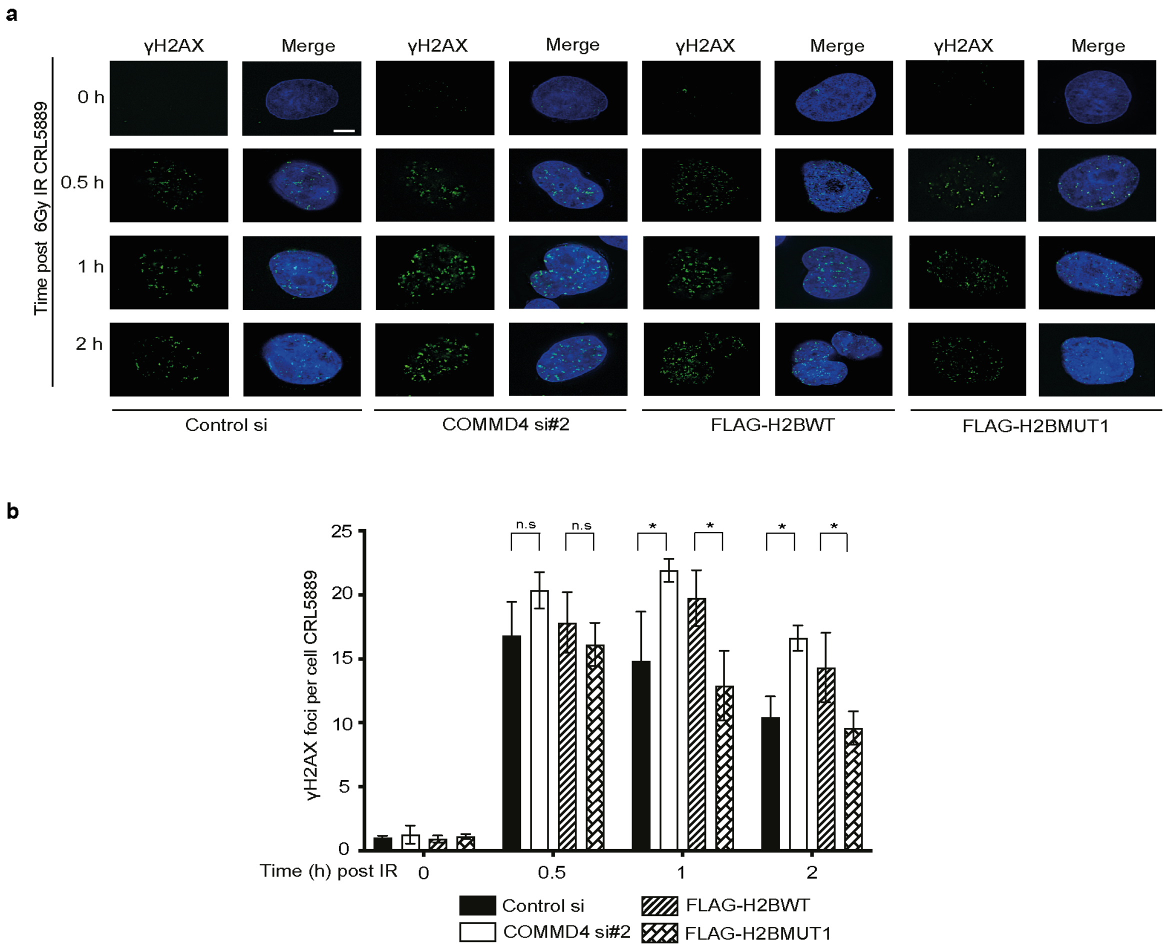

Fig. S4. Induction of γH2AX foci in CRL5889 cells. (a). Immunofluorescence staining of CRL5889 NSCLC cells transfected with control or COMMD4 siRNA #2 and FLAG-H2BWT or FLAG-H2BMUT1, treated with 6 Gy IR followed by recovery at time points shown. **(b)**. Plot of γH2AX foci numbers for **(a)**. n.s; not significant and *; $P < 0.05$. Error bars represent mean ± SD from three independent experiments where 50 cells were quantified per condition. γH2AX is shown in green and DAPI shows the nucleus. Scale bar denotes 5 μm.
