# Supplementary figures and images for: Targeting the COMMD4–H2B protein complex in lung cancer
Source: Br J Cancer. 2023 Nov 1;129(12):2014–24. doi: 10.1038/s41416-023-02476-8 (PMC10703884; doi:10.1038/s41416-023-02476-8)

Original western blot images

Figure 1

e

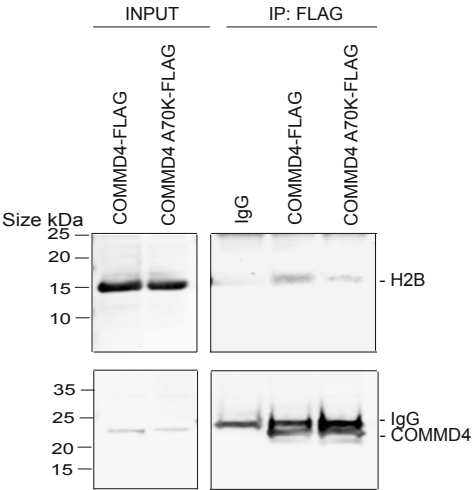

**Figure 2**

**a**

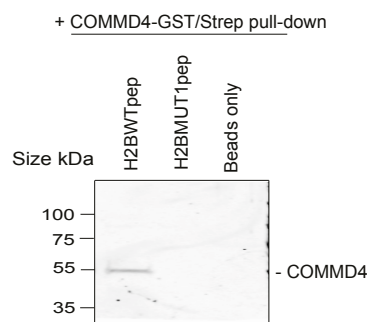

**b**

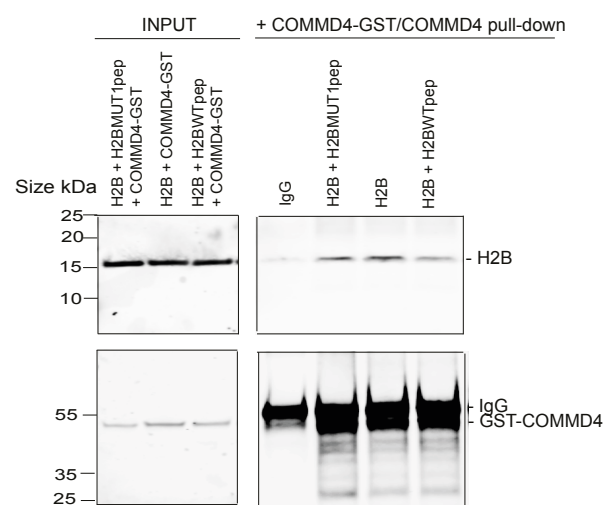

**c**

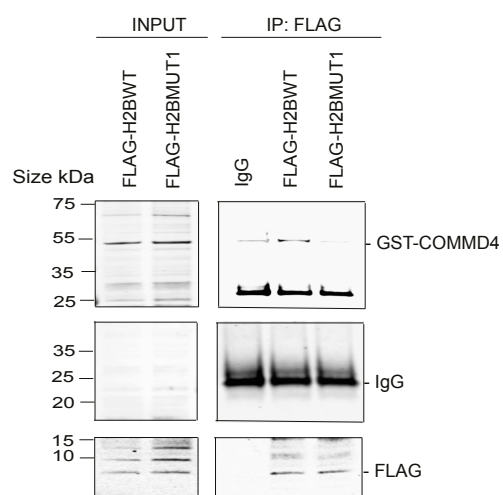

**Figure 3**

**a**

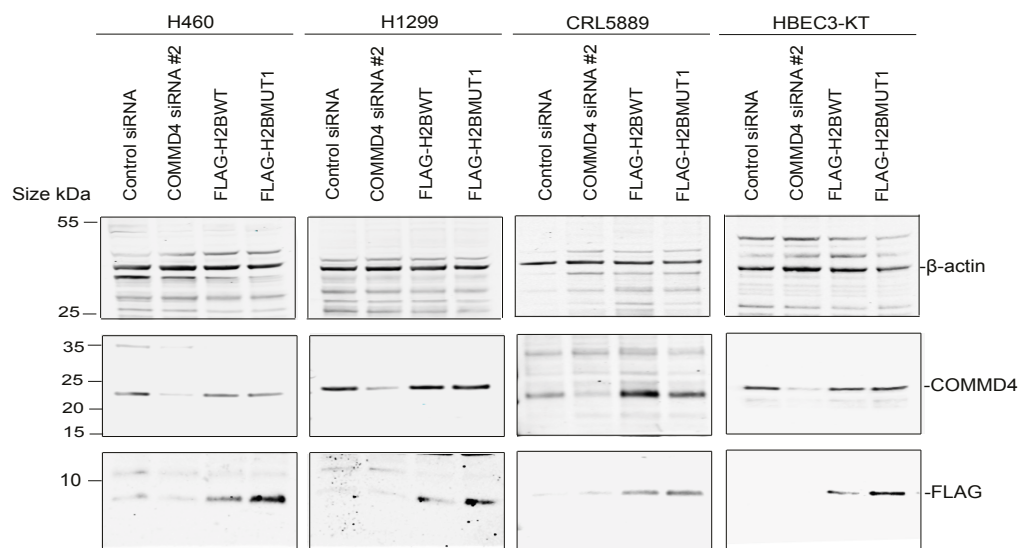

## Supplementary Figure 1

**b**

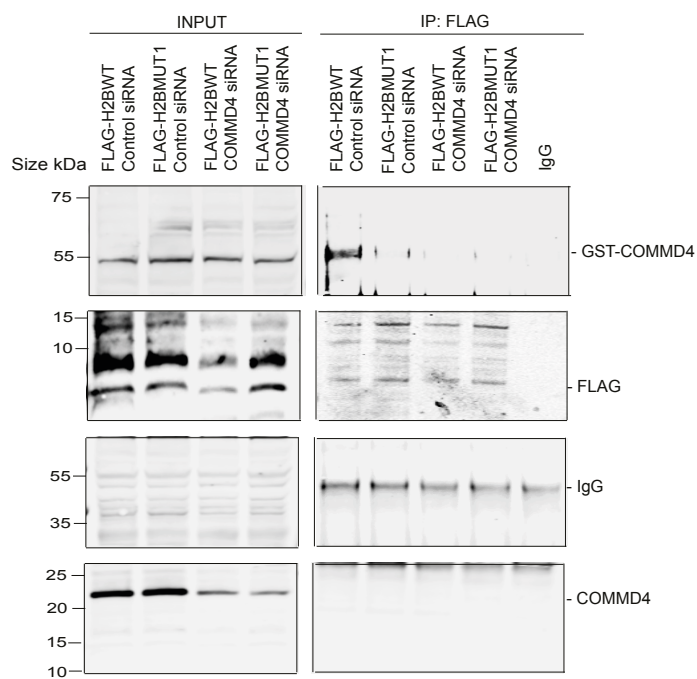

Supplement: Supplementary file 2 — Original uncropped western blots [file 41416_2023_2476_MOESM2_ESM.pdf]
